# Supplementary figures and images for: Strengthening research networks: Insights from a clinical research network in Brazil
Source: PLoS One. 2024 Aug 1;19(8):e0307817. doi: 10.1371/journal.pone.0307817 (PMC11293707; doi:10.1371/journal.pone.0307817)

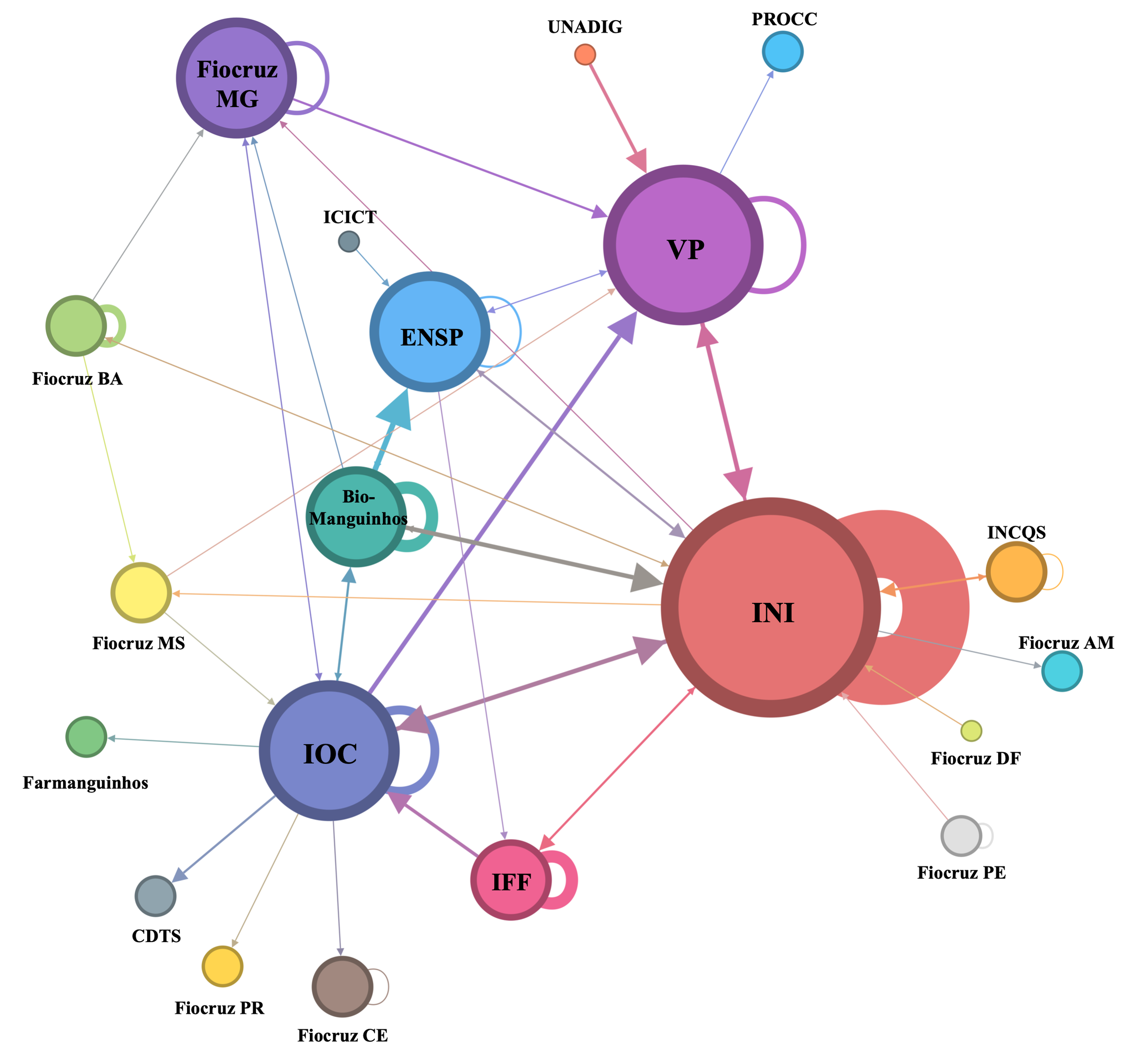

Supplement: S1 Fig — The circles (nodes) represent technical-scientific units, and the links between them represent a direct relationship from the respondent’s technical-scientific unit to his/her primary contacts. The size of a node is proportional to its indegree centrality. Auto-loops indicate that some professionals have identified a colleague from the same unit as their primary contact. Evandro Chagas National Institute of Infectious Diseases (INI); Fernandes Figueira National Institute for Women’s, Children’s and Adolescent Health (IFF); Vice-Presidency (VP); Oswaldo Cruz Institute (IOC); National School of Public Health (ENSP); Scientific Computing Program (PROCC); National Institute for Quality Control in Health (INCQS); Covid-19 Diagnostic Support Unit (UNADIG); Institute of Immunobiological Technology (Bio-Manguinhos); Institute of Drug Technology (Farmanguinhos); Center for Technological Development in Health (CDTS); Institute of Scientific and Technological Communication and Information in Health (ICICT); Fiocruz Minas Gerais (Fiocruz MG); Fiocruz Mato Grosso do Sul (Fiocruz MS); Fiocruz Paraná (Fiocruz PR); Fiocruz Bahia (Fiocruz BA); Fiocruz Ceará (Fiocruz CE); Fiocruz Pernambuco (Fiocruz PE); Fiocruz Distrito Federal (Fiocruz DF); Fiocruz Amazonas (Fiocruz AM). (TIF) [file pone.0307817.s001.tif]
