# Supplementary material for: Strengthening research networks: Insights from a clinical research network in Brazil
Source: PLoS One. 2024 Aug 1;19(8):e0307817. doi: 10.1371/journal.pone.0307817 (PMC11293707; doi:10.1371/journal.pone.0307817)
Supplement: S1 Table — (DOCX) [file pone.0307817.s002.docx]

**S1 Table: Metrics of the RFPC communication network**

| **Metrics** | **Value** |
| --- | --- |
| Number of nodes | 151 |
| Number of links | 187 |
| Size of the giant component | 137 (90.7%) |
| Network diameter | 5 |
| Network density | 0.008 |
