# Supplementary material for: Strengthening research networks: Insights from a clinical research network in Brazil
Source: PLoS One. 2024 Aug 1;19(8):e0307817. doi: 10.1371/journal.pone.0307817 (PMC11293707; doi:10.1371/journal.pone.0307817)
Supplement: S2 Table — (DOCX) [file pone.0307817.s003.docx]

**S2 Table: E-I index for Fiocruz technical-scientific units**

| **Units** | **Total connections** | **Internal connections** | **External connections** | **EI-index** |
| --- | --- | --- | --- | --- |
| Fiocruz CE | 1 | 1 | 0 | -1 |
| INI | 77 | 61 | 16 | -0.584 |
| IFF | 15 | 11 | 4 | -0.467 |
| Fiocruz BA | 10 | 7 | 3 | -0.400 |
| Fiocruz PE | 3 | 2 | 1 | -0.333 |
| Fiocruz MG | 7 | 4 | 3 | -0.143 |
| Bio-Manguinhos | 22 | 11 | 11 | 0 |
| Vice-Presidency (VP) | 11 | 5 | 6 | 0.091 |
| IOC | 24 | 8 | 16 | 0.333 |
| INCQS | 3 | 1 | 2 | 0.333 |
| ENSP | 7 | 2 | 5 | 0.429 |
| UNADIG | 3 | 0 | 3 | 1 |
| Fiocruz MS | 2 | 0 | 2 | 1 |
| Fiocruz DF | 1 | 0 | 1 | 1 |
| ICICT | 1 | 0 | 1 | 1 |
| Overall | 187 | 113 | 74 | -0.209 |

Evandro Chagas National Institute of Infectious Diseases (INI); Fernandes Figueira National Institute for Women's, Children's and Adolescent Health (IFF); Vice-Presidency (VP); Oswaldo Cruz Institute (IOC); National School of Public Health (ENSP); Scientific Computing Program (PROCC); National Institute for Quality Control in Health (INCQS); Covid-19 Diagnostic Support Unit (UNADIG); Institute of Immunobiological Technology (Bio-Manguinhos); Institute of Drug Technology (Farmanguinhos); Center for Technological Development in Health (CDTS); Institute of Scientific and Technological Communication and Information in Health (ICICT); Fiocruz Minas Gerais (Fiocruz MG); Fiocruz Mato Grosso do Sul (Fiocruz MS); Fiocruz Paraná (Fiocruz PR); Fiocruz Bahia (Fiocruz BA); Fiocruz Ceará (Fiocruz CE); Fiocruz Pernambuco (Fiocruz PE); Fiocruz Distrito Federal (Fiocruz DF); Fiocruz Amazonas (Fiocruz AM).
